# Müllerian Mimicry as a Result of Codivergence between Velvet Ants and Spider Wasps

**DOI:** 10.1371/journal.pone.0112942

**Published:** 2014-11-14

**Authors:** Juanita Rodriguez, James P. Pitts, Carol D. von Dohlen, Joseph S. Wilson

**Affiliations:** 1 Department of Biology, Utah State University, Logan, Utah, United States of America; 2 Department of Biology, Utah State University - Tooele, Tooele, Utah, United States of America; Federal University of Viçosa, Brazil

## Abstract

Recent studies have delineated a large Nearctic Müllerian mimicry complex in *Dasymutilla* velvet ants. *Psorthaspis* spider wasps live in areas where this mimicry complex is found and are phenotypically similar to *Dasymutilla*. We tested the idea that *Psorthaspis* spider wasps are participating in the *Dasymutilla* mimicry complex and that they codiverged with *Dasymutilla*. We performed morphometric analyses and human perception tests, and tabulated distributional records to determine the fit of *Psorthaspis* to the *Dasymutilla* mimicry complex. We inferred a dated phylogeny using nuclear molecular markers (28S, elongation factor 1-alpha, long-wavelength rhodopsin and wingless) for *Psorthaspis* species and compared it to a dated phylogeny of *Dasymutilla.* We tested for codivergence between the two groups using two statistical analyses. Our results show that *Psorthaspis* spider wasps are morphologically similar to the *Dasymutilla* mimicry rings. In addition, our tests indicate that *Psorthaspis* and *Dasymutilla* codiverged to produce similar color patterns. This study expands the breadth of the *Dasymutilla* Müllerian mimicry complex and provides insights about how codivergence influenced the evolution of mimicry in these groups.

## Introduction

Müllerian mimicry refers to the phenomenon in which sympatric, harmful species share a similar warning signal for mutual benefit against predation [1], [2]. This kind of mimicry has been well documented for several tropical groups, such as *Heliconius* butterflies [2]–[6] and poisonous Dendrobatidae and Mantellidae frogs [7]–[9]. Recently, a large Nearctic Müllerian mimicry complex was described in diurnally foraging *Dasymutilla* velvet ants (Hymenoptera: Mutillidae) [10]. These aposematic solitary wasps have wingless females that inflict a painful sting, which is effective as a defense against predators [10]. Although several Batesian mimics of velvet ants have been reported [11]–[15], the possibility that other harmful species might be Müllerian mimics of velvet ants has not been investigated.

Nine spider wasps in the genus *Psorthaspis* (Pompilidae) closely resemble velvet ant color patterns [16], and thus might be participating in the velvet ant mimicry complex. Because spider wasps are defended with a sting that invokes some of the most intense, instantaneous pain among stinging insects [17], and velvet ants and *Psorthaspis* spider wasps are attacked by some of the same predators (i.e., frogs, lizards and mammals) [18]–[22], *Psorthaspis* spider wasps and velvet ants could be Müllerian mimics of each other. However, the resemblance of *Psorthaspis* spider wasps to velvet ants, and the potential fit of both wasps to the same mimicry complex have never been quantified.

In the well-studied *Heliconius* Müllerian mimicry systems, codivergence, or the parallel divergence of ecologically associated, but unrelated, lineages, has been a major contributor to the development of numerous mimicry rings [23]. Codivergence has been proposed as some of the strongest evidence for coevolution [23]–[26]. Codivergence patterns alone, however, are not enough to demonstrate coevolution in the strict sense (i.e., evolution that occurs in populations of at least two species as the result of reciprocal selective influence) because selective pressures are often not measured between the two groups [23]. Although codivergence and the associated phenotypic convergence has been tested in some mimicry systems, investigations into the evolution of mimetic patterns in other systems, such as *Psorthaspis* spider wasps and velvet ants, have the potential to better illuminate the role of coevolution in the development of large Müllerian mimicry complexes.

Here, we investigate the phenotypic and phylogenetic similarities of *Dasymutilla* velvet ants and *Psorthaspis* spider wasps to address the following questions. 1) How well do *Psorthaspis* spider wasps fit in the described velvet ant mimicry rings? 2) Are the color pattern similarities between these wasp groups a result of codivergence?

## Methods

### Study system

Velvet ants and spider wasps are both classified as stinging wasps (Aculeata: Hymenoptera), and are both solitary parasitoids. Insect parasitoids are a special case of parasitic organisms because they ultimately kill their hosts during development [27]. Velvet ants are usually external parasitoids on the larvae or pupae of bees and solitary wasps. Their females are wingless, while males are typically winged and capable of flight [28]. There are more than 150 species of *Dasymutilla* velvet ants. Spider wasps (Pompilidae) are parasitoids of spiders. Both males and females are winged. There are 29 species of *Psorthaspis* spider wasps. These spider wasps use trapdoor spiders of the family Ctenizidae as hosts [29]. Even though the venom is primarily used to paralyze the host, the sting of both spider wasps and velvet ants also can be a deterrent to predation [10], [17].

### Morphometric analysis of color patterns

We quantified the color patterns of *Psorthaspis* using digital images following the procedure described by Wilson et al. [10], with the exception of setal characters, as they are not comparable between velvet ants and spider wasps. Characters included the percent black of the metasoma, integument color, and non-black metasomal color measured in red, green and blue (RGB). The color pattern of all the *Psorthaspis* species putatively involved in the mimicry complex was studied. Because there is some degree of intraspecific variation in color and pattern characteristics in spider wasp species, a representative individual was selected for each *Psorthaspis* species, on which measurements were made. These representative individuals were selected after the examination of over 1,000 specimens from 15 insect museums from five countries. All area and percentage measurements were made using the program ImageJ (http://rsb.info.nih.gov/ij/). Morphological characters were analyzed together with the data from Wilson et al. [10] using resemblance matrices, nonmetric multidimensional scaling (NMDS) based on a Bray-Curtis distance matrix, and permutational multivariate analysis of variance (PERMANOVA, [30]) in R [31] using the adonis function in the vegan package. The data gathered for this analysis are available on Figshare.

### Human perception of mimetic fidelity

Mimetic fidelity in Müllerian mimicry systems represents how well a given species matches a group of species (i.e., the mimicry ring). To measure mimetic fidelity of spider wasps involved in described Müllerian mimicry rings [10], we used methods outlined by Wilson et al. [32] for human perception tests. Even though many researchers are hesitant about the adequacy of using human rankings to establish mimetic fidelity, various studies have shown that human rankings are consistent with those of multivariate analyses of morphological data and avian response rankings [33]–[35]. Although human perception has been used mainly in systems where predators are birds [33], [36], other vertebrate predators like lizards have similar color vision to birds and humans [37], and may perceive prey them same way.

We presented slides showing an individual *Psorthaspis* species compared to all members of the velvet ant mimicry ring to which the species was most similar. Volunteers (N = 35) were directed to rank each *Psorthaspis* species on how well it fit into the associated mimicry ring. Rankings were based on a scale of 1 (very poor mimic) to 10 (excellent mimic). We included images of all the *Psorthaspis* species putatively included in the mimicry complex. All images were presented at magnifications such that all wasps had the same projected body length. Each slide was presented for 20 seconds following the protocols used by other similar studies [32], [33]. The mimetic fidelity of each spider wasp was estimated based on the mean score of a wasp compared to its assigned mimicry ring.

All volunteers participating in this study were students in lower division Biology courses at Utah State University–Tooele. Students were presented with a short presentation introducing the concepts of Batesian and Müllerian mimicry and were then given the option to participate in a survey designed to rank mimetic fidelity of wasps. If students agreed to participate, they were given a link to the website containing the survey. To our knowledge, the volunteers were not experts in insect identification. This effectively resulted in mimetic fidelity scores that were based on overall resemblance of a mimic to a mimicry ring rather than on preconceived ideas of what specific parts of a mimic should match the ring. All participants were over the age of 18, and no data relating to the volunteers were gathered. No approval from the university was requested for this research because no information about living individuals was collected (*i.e.,* the research did not involve human subjects as per the Code of Federal Regulations 45 CFR part 46). Volunteers were simply used to gather information concerning morphological similarities between the insects involved in this study. Because of the need to protect the anonymity of our volunteers, no questions were asked regarding any physical characteristics that would affect ranking mimics and models (i.e., colorblindness). While this potentially could influence the reported mimetic fidelity scores, we think any influence of colorblindness would be minimal, due to the nature of aposematic signals in spider wasps and velvet ants. These warning signals primarily result from contrasting black and red or yellow patterns, which would still be visually distinct to colorblind individuals.

### Estimation of geographical distribution

To determine the distribution of each of the *Psorthaspis* color patterns identified in this study we geo-referenced 1,032 *Psorthaspis* specimens, from all mimic species, from 13 natural history collections and downloaded data on geo-referenced *Psorthaspis* specimens in the Southwest Collections of Arthropods Network (SCAN) [38]. We manually plotted the collection localities of each species on a map using the software Google Earth 5.0 (http://earth.google.com) and estimated geographic distributions by drawing a line encompassing all of the collection localities. These estimated distributions were visually compared to the distributions of velvet ant mimicry rings published by Wilson et al. [10]. The data points used for this analysis are available on Figshare.

### Molecular data and phylogenetic inference

We compiled a data set of four genes (28S, elongation factor 1-alpha, wingless, and long-wavelength rhodopsin) for 13 *Psorthaspis* species and one outgroup (*Aporus idris*), which were previously published by Rodriguez et al. [39]. Two of the putative mimic species, *Psorthaspis nigriceps* and *Psorthaspis texana* could not be included because of the lack of suitable molecular data. Sequences were aligned using Geneious Alignment in Geneious 5.4 [40], and manually refined. The model of molecular evolution used for each gene and by codon position was the same used by Rodriguez et al. [39] except for introns from long-wavelength rhodopsin, for which the model was determined in MrModelTest [41]. Single-gene phylogenies were estimated through a Bayesian framework implemented in MrBayes 3.2 [42] to check for potential conflict between gene trees. Single-gene matrices were then concatenated using Geneious 5.4 to produce a combined matrix, using the best partition scheme used by Rodriguez et al. [39], and an additional partition including long-wavelength rhodopsin introns with the model GTR+I+G. MCMC chains were run for 10,000,000 generations, with sampling every 1,000 generations. Effective sample size (ESS), burn-in, and graphical examination of chain convergence were examined in Tracer 1.5 [43].

A chronogram of *Psorthaspis* was inferred from the combined matrix in a Bayesian framework using BEAST 1.7.5 [44] under an uncorrelated lognormal relaxed-clock model [45], [46]. Substitution models were unlinked among partitions; the underlying clock and trees were linked. The crown-group node of all *Psorthaspis* was assigned a normal prior of mean = 12.9 Ma (SD = 10), based on results of Rodriguez et al. [39]. Two separate Markov Chain Monte Carlo (MCMC) searches were performed for 10,000,000 generations. Effective sample size (ESS) and graphical chain convergence were examined in Tracer 1.5. Independent runs were assembled with LogCombiner 1.7.5. and 10% of the generations were discarded as burn-in. Divergence time estimations of *Dasymutilla* were obtained from Williams [28].

### Codivergence test

To determine if there was codivergence between *Dasymutilla* and *Psorthaspis* mimicry rings we performed two permutation analyses in R using the phylogenetic trees of both groups. First, an analysis that calculates the Pearson’s correlation coefficient [47] was implemented using the correlation between the distances of the two phylogenies. Second, we applied an analysis that calculates the ParaFitGlobal statistic [48], which uses transformed distances derived from the phylogenetic trees into matrices of principal coordinates. Both analyses test the null hypothesis that the two groups are evolving independently. We performed 100,000 simulations for both tests. Additionally, we constructed a tanglegram linking phenotypically similar species between the phylogenies of *Dasymutilla* and *Psorthaspis*. The tanglegram was created using the function “cophyloplot” from the Ape package in R. This function does not optimize the tanglegram and rather is just a visual representation of the shared branching events.

## Results

### Morphological results

**Figure 1:**
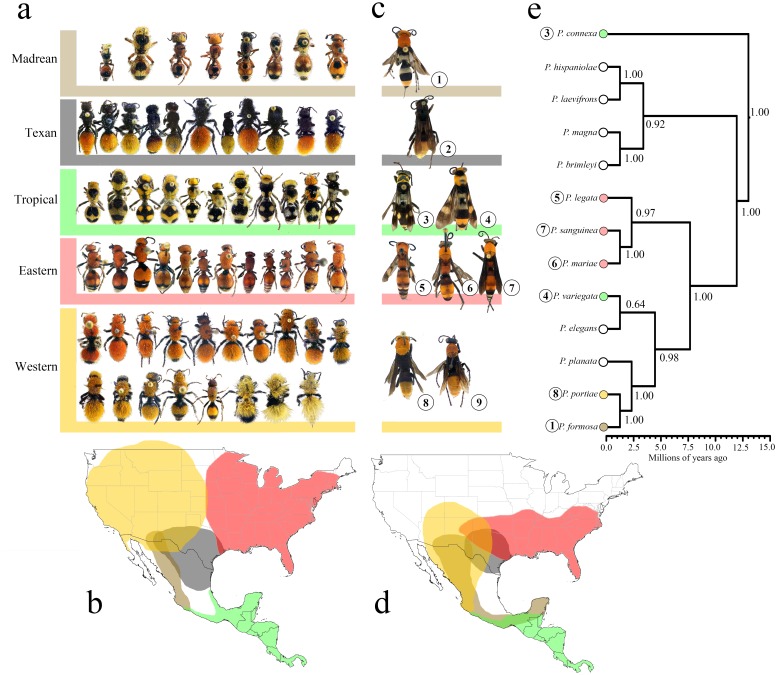
*Psorthaspis* spider wasp and velvet ant mimicry ring morphology and distribution, and *Psorthaspis* chronogram (a) Color patterns of the five velvet ant mimicry rings described by Wilson et al. (2012). (b) Geographic distribution of the five velvet ant mimicry rings. (c) Color pattern of the nine *Psorthaspis* species placed next to their putative velvet ant mimicry rings. Numbers under each *Psorthaspis* species correspond to their positions on the phylogenetic tree and in Figure 2. Species number 2 [*Psorthaspis texana*] and number 9 [*Psorthaspis nigriceps*] did not yield usable DNA samples and was therefore not included in the phylogenetic analysis. (d) Geographic distributions of the *Psorthaspis* spider wasp mimicry rings. (e) *Psorthaspis* spider wasp chronogram. Bayesian posterior probabilities are displayed on nodes.

**Figure 2:**
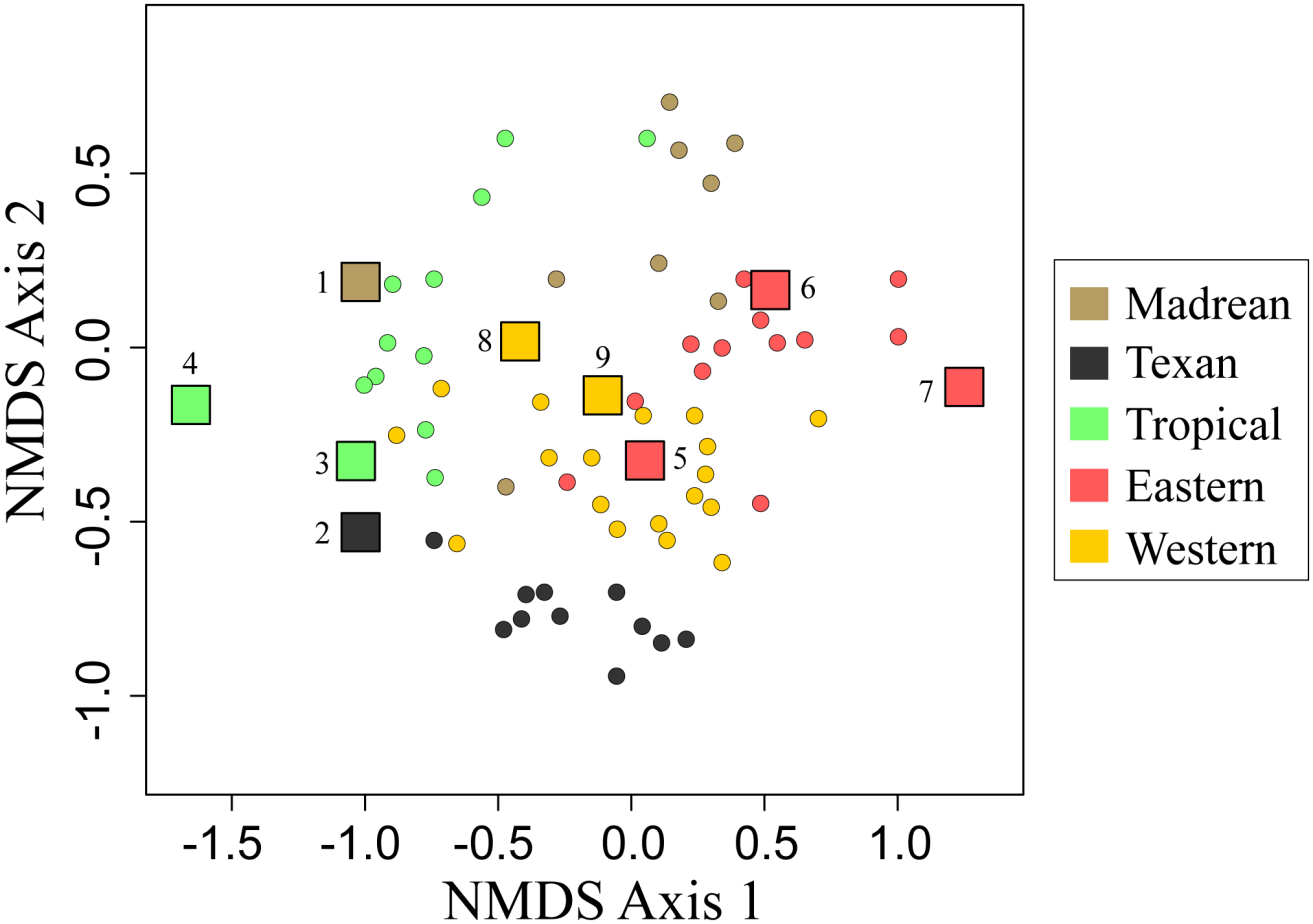
Morphological trait NMDS ordination plot of *Psorthaspis* spider wasps and the *Dasymutilla* mimicry rings to which they were assigned *a priori.* Circles denote velvet ant data (from Wilson et al. 2012) and squares represent *Psorthaspis* data. Numbers represent *Psorthaspis* species numbered in Figure 1.

The NMDS and PERMANOVA analyses indicate that morphological traits of *Psorthaspis* spider wasps fall within *Dasymutilla* mimicry rings to which they were assigned *a priori* (Figures 1 and 2). The overall effect of the mimicry ring as a categorical variable was F = 22.503, R^2^ = 0.616, NMDS stress = 0.14, P<0.001. Despite the overall similarity, the plot of the NMDS and the stress value show that *Psorthaspis* often do not fit tightly with *Dasymutilla* in morphospace, but rather seem to fall out near the periphery of the velvet ant clusters. The sole exception was the Eastern mimicry ring, which fell within the middle of the velvet ant distribution (Figure 2).

Mimetic fidelity reported by volunteers was more variable for spider wasps (Table 1) than for velvet ants [32]. Although some spider wasps received mimetic fidelity scores comparable to the velvet ants (e.g., the Tropical, Madrean and Eastern mimicry rings), others received much lower scores (e.g., the Western and Texan mimicry rings).

**Table 1:** Human perception tests of mimetic fidelity of *Psorthaspis* species reported by volunteers (N = 35).

Spider wasp species	Average mimetic fidelity score	SD	Assigned mimicry ring
***P. formosa***	4.60	2.19	Madrean
***P. texana***	4.71	3.18	Texan
***P. connexa***	8.74	1.52	Tropical
***P. variegata***	6.29	2.53	Tropical
***P. legata***	8.83	1.69	Eastern
***P. mariae***	6.74	2.17	Eastern
***P. sanguinea***	6.63	2.17	Eastern
***P. portiae***	5.26	2.13	Western
***P. nigriceps***	5.89	1.91	Western

Average mimetic fidelity of each spider wasp species indicates how well each species matches the velvet ant mimicry ring it was phenotypically and geographically most similar to. Scores are based on a scale of 1 (very poor mimic) to 10 (excellent mimic).

### Geographical overlap between *Psorthaspis* and *Dasymutilla* mimicry rings

Distributions of *Psorthaspis* spider wasp and *Dasymutilla* velvet ant species putatively involved in the same mimicry rings are largely congruent (Figure 1). In general, *Dasymutilla* mimicry rings have a more widespread distribution than that of spider wasps, particularly in northern latitudes. Distributions of *Psorthaspis* mimicry rings show much greater overlap with each other than do those of *Dasymutilla* velvet ants (Figure 1). This is particularly apparent in the distribution of the *Psorthaspis* Madrean mimicry ring, which is geographically larger than the Madrean ring in *Dasymutilla*. Similarly, the Western *Psorthaspis* ring extends farther south than the Western *Dasymutilla* ring, resulting in a larger overlap between *Psorthaspis* Western and Madrean rings. In addition, the Texan *Psorthaspis* ring seems to be more restricted than its *Dasymutilla* counterpart (Figure 1).

### Phylogenetic relationships, divergence times and codivergence results

The phylogeny of *Psorthaspis* suggests that mimetic species do not compose a monophyletic group. Divergence time estimates suggest that the common ancestor of extant *Psorthaspis* species arose ca. 12.9 Ma (CI = 8.76,18.02). Because taxa composing the sister group to *Psorthaspis* (i. e. species of *Allaporus*) are non-mimics [39], it is probable that mimicry arose in *Psorthaspis* after it diverged from its sister group ca. 18.14 Ma (CI = 13.28,23.71). The origin of *Dasymutilla* was ca. 21 Ma (CI = 18,23), and the divergence from its sister group was 23 Ma (CI = 21,27) (Williams 2012); therefore, the origin of mimicry in *Dasymutilla* was likely 23 Ma or later. The codivergence tests suggest topological concordance between the lineages representing mimicry rings of *Psorthaspis* and *Dasymutilla* (Pearson’s p = 0.0027, ParaFitGlobal p = 0.047). The tanglegram of *Psorthaspis* and *Dasymutilla*, is somewhat complicated by the lack of order of mimetic color patterns in *Dasymutilla*. Even though at a first glance the phylogenies compared do not have obvious shared branching patterns (due partially to the random distribution of color characters on the velvet ant phylogeny [10]), statistical tests are often a more powerful way to detect correlation because, besides cospeciation, other types of events can be taking place, like independent speciation, and extinctions. Because of this, even host-parasite phylogenies are only rarely completely congruent [47].

## Discussion

### Fit of *Psorthaspis* to the velvet ant mimicry rings

Assessing the strength of the fit of mimics to their model is challenging; therefore, we used multiple lines of evidence to support our results. Results of the morphometric analyses and human perception tests indicate that *Psorthaspis* spider wasps likely participate in the *Dasymutilla* velvet ant mimicry complex, albeit with a lower mimetic fidelity than the velvet ant participants, which suggests some degree of imperfect mimicry. This lower fidelity of the spider wasps is not surprising, given the many morphological differences between the two groups (e.g., wings, setae, etc.). The lower mimetic fidelity might also be explained by the broad geographic overlap in some *Psorthaspis* mimicry rings. Such overlap between adjacent mimicry rings is correlated with lower mimetic fidelity in velvet ants [32], and likely accounts for lower mimetic fidelity in spider wasps as well.

### Evidence for coevolution

**Figure 3:**
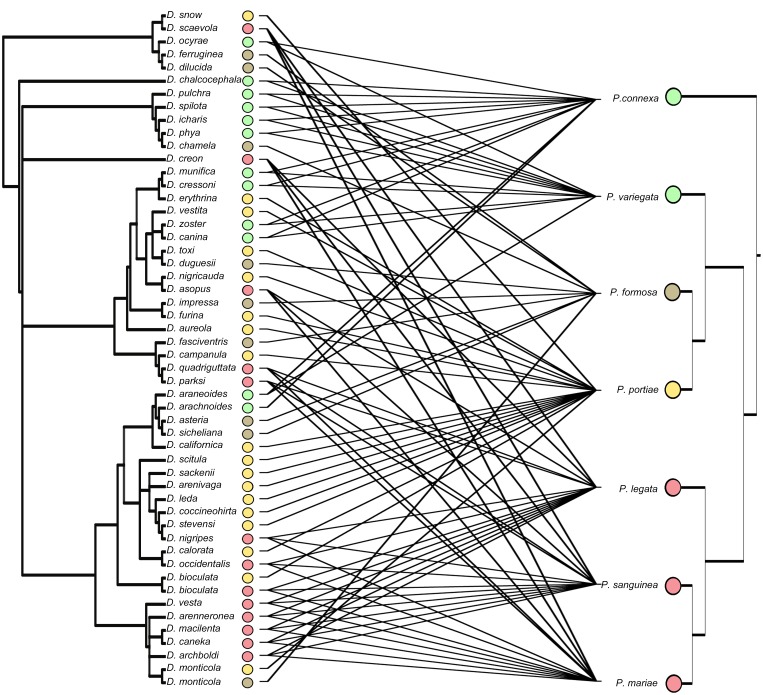
Tanglegram of *Psorthaspis* (left topology) and *Dasymutilla* (right topology). Lines connect between members of the same mimicry rings in the two groups.

While not tested directly in this study, our results suggest that coevolution played a role in the development of the large velvet ant and spider wasp mimicry complex. Several lines of evidence (i.e., morphological similarity, shared geographic distribution, codivergence) support this assertion. First, while it is not immediately evident from the topologies of the *Dasymutilla* and *Psorthaspis* phylogenies (Figure 3), statistical tests show evidence of codivergence between mimetic lineages of the two wasp families. This suggests that the evolution of mimicry between these wasp groups must have involved convergence at the genetic and phenotypic level, such as has been found for Neotropical butterflies [49], [50].

Molecular dating estimates suggest that *Dasymutilla* likely evolved approximately 5 Ma earlier than *Psorthaspis*, although there is some overlap in the CI estimates of the two groups. This would suggest that the similar color patterns of *Psorthaspis* spider wasps and *Dasymutilla* velvet ants likely are the result of codivergence (Figure 1). Interestingly, the low fidelity of spider wasp mimicry is not equal across all mimicry rings. For example, *Psorthaspis* participating in the Tropical mimicry ring received higher fidelity scores than many of the mimicry rings in higher latitudes (Table 1). This supports the hypothesis that tropical mimics converge on precise mimicry, whereas temperate mimics seem to converge on an “impressionistic” or more relaxed pattern [51]. It also supports the hypothesis that mimicry rings that are more isolated (have little geographic overlap with adjacent mimicry rings) tend to have higher mimetic fidelity because the ecological community is more uniform in coloration, which can lead stronger convergence on one color pattern [32]. The Tropical mimicry ring of *Psorthaspis* has the least amount of distributional overlap with other mimicry rings, which might explain their high mimetic fidelity.

Coevolution involves reciprocal selective pressures between two groups. While not tested directly, reciprocal selective pressures between *Psorthaspis* spider wasps and *Dasymutilla* velvet ants may indeed be taking place. These two wasp groups share predators [18]–[22], and while *Dasymutilla* velvet ants likely evolved aposematic coloration before *Psorthaspis* spider wasps, once spider wasps converged phenotypically, the aposematic signal of velvet ants would be strengthened because of the presence of harmful, aposematic co-mimics (spider wasps). Likewise, the spider wasp aposematic coloration would also be strengthened through the presence of their harmful aposematic co-mimics (velvet ants). Thus, both groups would be imposing coevolutionary selective pressures on each other.

### Summary

We provide evidence that *Psorthaspis* spider wasps participate in velvet ant mimicry rings. Furthermore, we find evidence that the two groups codiverged to produce a similar color pattern. This study expands the breadth of the largest known North American Müllerian mimicry complex to include spider wasps as well as velvet ants. This large mimicry complex is an intriguing system that should be the focus of further investigations into the evolution of predator avoidance strategies in the temperate regions, the evolution of aposematic coloration, and the evolution of Müllerian mimicry involving unrelated taxa.
